# Recurrent or junctional lumbar foraminal herniated disc in patients operated with trans pars microscopic approach

**DOI:** 10.1007/s10143-023-02109-x

**Published:** 2023-08-29

**Authors:** Matteo Monticelli, Clarissa Ann Elisabeth Gelmi, Alba Scerrati, Michele Alessandro Cavallo, Pasquale De Bonis

**Affiliations:** Neurosurgery Unit, Department of Translational Medicine and for Romagna, Ferrara University, Ferrara, Italy

**Keywords:** Trans pars microscopic technique, Foraminal herniated lumbar disc, Junctional herniated lumbar disc, Recurrences of foraminal herniated lumbar disc, Minimally invasive spine surgery

## Abstract

This is a retrospective monocentric study. The aim of this study is to analyze the incidence of recurrent or junctional lumbar foraminal herniated disc, in patients treated with trans pars microsurgical approach. Foraminal lumbar disc herniation represents a challenging pathology for the spinal surgeon. The appropriate surgical approach still represents a matter of debate. Several open and minimally-invasive techniques have been developed, in order to allow a proper tissue exposure and preserving the vertebral stability. The trans pars approach has already been described as a possible alternative, allowing excellent exposure of the herniated fragment with minimum bone removal. While few studies have analyzed the very low rate of post-operative instability, no articles deal with the incidence of post-operative herniated disc recurrence or junctional disc herniation in patients treated with this technique. We enrolled 160 patients operated at our institution. A univariate and multivariate analysis of possible factors influencing outcome (age, sex, level and BMI) was performed. Outcome variables were recurrent or junctional herniated disc. At the end, 135 patients were analyzed. Of the 135 patients, six presented recurrent herniated disc (4.4%) and other three developed a junctional herniation (2.2%). The occurrence of junctional herniated disc or recurrent herniated disc was not influenced by the analyzed variables, both at univariate and at multivariate analyses. The trans pars approach presents a low rate of recurrence and junctional herniation. Age, sex, level, and BMI do not influence the recurrence rate, both at same level and at junctional level.

**Supplementary Information:**

The online version contains supplementary material available at 10.1007/s10143-023-02109-x.

## Introduction

Lumbar disc herniation (LDH)-related back pain still represents the most common pathological condition that the spine surgeon faces in his or her clinical practice [1–3], and among them, lumbar foraminal disc herniations (FLDH) are undoubtedly the case that results in the most severe pain for the patient and the greatest likelihood of neurological deficits [4]. Over time, several surgical approaches have been proposed for their treatment most of which involve massive bony removal so as to achieve wider exposure at the expense, however, of potentially undermining the stability of the spine [5, 6].

In order to minimize vertebral instability, relatively new surgical techniques focused on obtaining maximum exposure with minimal bone removal have been proposed.

The trans pars interarticularis approach is one of them, and its main focus is at exposing the herniated fragment in the foramen of conjugation by partial removal of the vertebral isthmus alone without touching the joint in any way thus preserving the stability of the segment [7, 8].

This approach has been demonstrated to be safe and effective to treat FHLD [8].

However, evidences on possible recurrence of LDH or occurrence of a junctional LDH in patients with FLDH treated with the trans pars approach are limited.

A recent literature meta analysis [9] shows how the recurrence rate of nucleus pulpous herniation after discectomy is a wide common problem with rates ranging from 2 to 25% despite the surgical technique used in the absence of any work specifically analyzing the trans pars technique.

Similarly, at the present day, no recent studies in literature exist specifically dealing with junctional herniations after discectomies in general and with microscopic trans pars techniques in particular; the only existing studies are about adjacent segment pathology after lumbar arthrodesis [10–12].

The aim of this retrospective study is therefore to specifically analyze the rate of recurrent or junctional LDH after treating a FLHD with the microscopic trans pars approach and to show if patients’ characteristics such as age, sex, level, and BMI could influence this rate.

## Surgical technique

The trans pars approach is a microsurgical technique with the aim to give access to the foraminal region of interest (in order to decompress the nerve root, to perform a discectomy, treat the herniated disc syndrome, and prevent the relapse) in a less destructive manner compared to other microsurgical options available but achieving the same result in terms of efficacy.

The patient is under general anesthesia, prone, or in the knee-chest position.

A paramedian incision of approximately 3 cm must be carried out about 1cm far from midline; a dissection of the subcutaneous tissue from the underlying fascia must be performed, and therefore, the fascia is cut in the whereabouts of the lateral aspect of the spinous process.

A subperiostal dissection of the multifidus muscle must be carried out; the surgeon must carry on the dissection laterally to expose the inferior facet joint and the pars interarticularis.

The pars interarticularis (as known as isthmus) from the surgical view lies right above the intervertebral foramen, from which the nervous root corresponding to the upper vertebra transits. A dissector is then placed in the angle between the superior aspect of the inferior facet joint and the lateral aspect of the pars interarticularis as a radiopaque marker. The removal of the lateral aspect of the isthmus must be carried out using a twist-drill; also, a small part of the superior aspect of the inferior facet joint can be removed; the removal of the medial portion of the pars interarticularis is completed using Kerrison rongeurs. The surgeon has now visual on the lateral portion of the ligamentum flavum, which can be cut and removed.

As a result, the intraforaminal structures are now exposed: the nerve root lies usually cranially, while the disc space can usually be seen in the caudal part of the surgical window. Once the herniated disc into the foramen is identified, it must be gently and safely isolated from the nerve root then removed. Finally, a complete hemostasis of the site must be obtained and the fascia, the subcutaneous tissue, and the skin are closed in layers.

## Materials and methods

This is a retrospective study enrolling patients suffering from foraminal herniated lumbar disc (FLDH) and treated with microsurgical trans pars approach.

Local ethical committee approved the present study (55/2019/Oss/AOUFe).

### Patients

Demographic and pre- and postoperative clinical characteristics were recorded for all patients (age, sex, BMI), including pain assessment and nerve root palsy (length of symptomatic period, presence of motor deficit, and radicular pain before the operation) (Table 1).

**Table 1:** Demographic characteristics

ID PAT	Age	Sex	Weight (kg)	Height (cm)	BMI	Level	Side	Intervention duration (min)	Duration symptoms(months)	Recurrence	Recurrence time (months)
AA	71	M	82	175	26.78	L5-S1	Right	120	1	NO	
BD	47	M	93	189	26.04	L3-L4	Left	93	2	NO	
BR	65	M	63	163	23.71	L2-L3	Right	85	2	NO	
BAM	71	F	72	168	25.51	L4-L5	Left	18	2	NO	
BL	31	M	97	190	26.87	L5-S1	Left	70	3	NO	
BM	66	M	90	165	33.06	L4-L5	Right	100	3	NO	
BG	74	M	95	170	32.87	L4-L5	Left	35	0.5	NO	
CS	59	F	43	155	17.90	L3-L4	Left	60	1	NO	
CG	54	M	70	170	24.22	L4-L5	Right	85	12	NO	
CC	71	M	83	167	29.76	L4-L5	Right	65	2	NO	
CS	60	M	66	166	23.95	L4-L5	Left	80	3	NO	
CR	60	M	91	183	27.17	L4-L5	Left	60	2	NO	
FS	79	F	67	160	26.17	L3-L4	Left	150	2	YES	0
FE	59	M	88	180	27.16	L3-L4	Right	84	1	NO	
GR	66	M	90	178	28.41	L3-L4	Right	55	1	NO	
GE	52	M	85	170	29.41	L3-L4 e L4-L5	Right	150	1	NO	
GD	65	M	88	170	30.45	L3-L4	Both	75 (1)-80 (2)	2	NO	
GI	63	M	80	174	26.42	L5-S1	Right	85	3.5	NO	
GI1	47	M	63	168	22.32	L4-L5	Left	25	0.5	NO	
LF	46	M	92	173	30.74	L3-L4	Left	105	1	NO	
LL	86	F	46	155	19.15	L3-L4	Right	80	3	NO	
LG	75	F	63	162	24.01	L4-L5	Left	65	1	NO	
MM	70	M	77	182	23.25	L4-L5	Right	57	3	NO	
MM1	61	M	70	174	23.12	L4-L5	Left	50	3	NO	
MN	69	F	73	155	30.39	L3-L4	Left	80	0.5	NO	
MMG	62	F	100	175	32.65	L3-L4	Left	105	7	NO	
MD	70	F	78	154	32.89	L4-L5	Left	160	2	NO	
MD	55	M	98	187	28.02	L2-L3	Right		2	NO	
MS	68	M	80	170	27.68	L4-L5	Right	60	3	NO	
MA	81	M	86	169	30.11	L2-L3	Left	100	2	NO	
MD	72	M	60	165	22.04	L5-S1	Left	97	2	NO	
MC	66	F	78	165	28.65	L4-L5	Left	60	1	NO	
NE	57	M	72	170	24.91	L3-L4	Right	47	1	NO	
NC	57	F	70	160	27.34	L4-L5	Right	70	0.75	NO	
NG	50	M	64	170	22.15	L5-S1	Left	69	3	NO	
PS	63	M	75	170	25.95	L3-L4	Left	105	6	NO	
PM	53	M				L5-S1	Right	70	1	NO	
PG	62	F	78	170	26.99	L2-L3	Left	65	1	NO	
PA	75	F	73	160	28.52	L3-L4	Left	40	2.5	NO	
RL	81	M	82	170	28.37	L3-L4	Right	75	1	NO	
RS	42	M	122	178	38.51	L4-L5	Right	75	5	NO	
RD	60	F	55	160	21.48	L5-S1	Left	65	6	NO	
SE	78	F	58	160	22.66	L4-L5	Left	65	6	NO	
SE	69	M	70	170	24.22	L4-L5	Right	60	2.5	NO	
SM	46	M	63	170	21.80	L5-S1	Right	75	0.25	NO	
SS	57	M	64	180	19.75	L4-L5	Left	110	2	NO	
SR	57	F	56	167	20.08	L2-L3	Right	144	3	NO	
SG	62	M	66	175	21.55	L3-L4	Right	68	1.5	NO	
SM	57	M	74	178	23.36	L3-L4	Right	85	3	NO	
TL	51	M	100	180	30.86	L2-L3	Right	115	0.5	NO	
TG	64	M	83	180	25.62	L4-L5	Left	95	0.5	NO	
TL1	62	M	87	183	25.98	L3-L4	Right	40	1	NO	
TE	74	F	73	182	22.04	L2-L3	Left	55	0.5	NO	
VF	67	M	93	185	27.17	L3-L4	Right	85	5	NO	
ZF	60	M	105	173	35.08	L3-L4	Left	87	2	NO	
ZE	59	M				L2-L3	Left	105	1	NO	
ZI	80	M	56	155	23.31	L2-L3	Left	65	3	NO	
GL	56	M	57	155	23.73	L4-L5	Right	60	1.5	NO	
BM	61	F	64	160	25.00	L3-L4	Left	65	3	NO	
YT	68	M	74	170	25.61	L3-L4	Right	85	24	NO	
ZM	58	M	66	170	22.84	L5-S1	Left	59	2	NO	
FA	51	M	83	183	24.78	L2-L3	Left	80	0.5	NO	
SM	74	M	80	175	26.12	L2-L3	Right	39	0.25	NO	
SL	50	F	86	160	33.59	L4-L5	Right	79	1	NO	
ZR	63	M	77	171	26.33	L4-L5	Left	72	2	NO	
VR	53	M	80	178	25.25	L3-L4	Left	45	1	NO	
GV	61	F	64	164	23.80	L4-L5	Left	90	2	NO	
CM	59	F	80	162	30.48	L2-L3	Left	70	2	NO	
CP	79	M	60	165	22.04	L4-L5	Right	110	2	NO	
FR	47	M	67	164	24.91	L4-L5	Right	90	24	NO	
GS	51	M	67	163	25.22	L4-L5	Right	39	1.5	NO	
MG	69	M	90	174	29.73	L3-L4	Right	45	0.5	NO	
BP	71	M	74	168	26.22	L4-L5	Right	80	3	NO	
CR	48	F	85	159	33.62	L2-L3	Right	70	8	NO	
CC	49	M	93	178	29.35	L4-L5	Left	70	2	NO	
BR	51	F	80	159	31.64	L2-L3	Right	50	2	NO	
BF	49	F	60	165	22.04	L3-L4	Right	95	5	NO	
PG	76	M	88	176	28.41	L4-L5	Right	70	1	NO	
DM	42	M	75	166	27.22	L4-L5	Left	65	3	YES	18
CGL	53	M	80	182	24.15	L3-L4	Right	64	1	NO	
BI	70	M	62	169	21.71	L4-L5	Left	45	0.5	NO	
DL	70	M	86	180	26.54	L3-L4	Right	90	3	NO	
FG	67	M	72	161	27.78	L4-L5	Right	90	0.25	NO	
FV	58	M	80	176	25.83	L5-S1	Left	100	2	NO	
SL	60	M	90	177	28.73	L4-L5	Right	100	0.5	NO	
BM	48	F	52	175	16.98	L4-L5	Right	60	1	NO	
PG	47	M	63	173	21.05	L3-L4	Right	65	2	NO	
SL	56	M	110	178	34.72	L4-L5	Right	110	3	NO	
PG	60	M	100	173	33.41	L2-L3	Left	80	2	NO	
CA	52	F	72	172	24.34	L4-L5	Right	105	1	NO	
VM	48	M	65	190	18.01	L3-L4	Right	135	12	YES	4
CA	80	M	79	173	26.40	L4-L5	Right	160	12	NO	
IC	65	F	73	158	29.24	L4-L5	Right	16	2	NO	
CA	70	F	59	167	21.16	L4-L5	Left	70	12	NO	
CF	73	F	70			L4-L5	Left	130	3.5	NO	
RE	61	M	90	177	28.73	L3-L4	Right	90	2	NO	
VA	62	F	53	159	20.96	L3-L4	Right	58	12	NO	
AMA	64	F	65	161	25.08	L2-L3	Right	40	3	NO	
FP	68	M	80	175	26.12	L4-L5	Left	103	5	NO	
SR	73	M	101	183	30.16	L4-L5	Right	153	8	YES	7
SO	66	F	65	166	23.59	L4-L5	Right	43	3	NO	
DR	78	M	80	175	26.12	L4-L5	Right	35	2	NO	
ZE	25	M	76	178	23.99	L5-S1	Right	60	3	NO	
GB	81	M	63	160	24.61	L3-L4	Right	80	18	NO	
RMA	59	F	79	162	30.10	L4-L5	Left	94	2	NO	
MR	75	M	86	175	28.08	L4-L5	Left	85	7	NO	
SB	52	M	84	183	25.08	L3-L4	Right	168	1	NO	
PV	56	M	68	168	24.09	L5-S1	Right	50	4	YES	20
CA	54	F	70	170	24.22	L5-S1	Right	105	3	NO	
GV	70	M	102	183	30.46	L2-L3	Right	150	2	NO	
AN	52	F	88	168	31.18	L3-L4	Left	45	2	NO	
FM	51	M	91	181	27.78	L3-L4	Right	45	2	NO	
DAA	48	M	90	165	33.06	L5-S1	Left	85	12	NO	
PA	49	F	57	160	22.27	L4-L5	Right	34	94	NO	
ZD	66	F	61	158	24.44	L4-L5	Left	57	2	NO	
GI1	72	F	75	156	30.82	L3-L4	Left	35	7	NO	
PF	71	M	68	182	20.53	L4-L5	Right	40	1	NO	
CV	47	M	93	178	29.35	L4-L5	Right	100	3	NO	
FAM	63	F	80	160	31.25	L5-S1	Right	55	2	NO	
GM	57	M	78	182	23.55	L4-L5	Right	55	2	NO	
AG	80	M	62	165	22.77	L4-L5	Right	45	3	NO	
BG	67	M	80	165	29.38	L4-L5	Right	69	2	NO	
VC	54	M	105	186	30.35	L4-L5	Right	70	2	NO	
GR	67	F	77	170	26.64	L4-L5	Right	95	3	NO	
ZC	57	F	59	165	21.67	L4-L5	Left	30	3	NO	
FF	38	M	75	177	23.94	L5-S1	Right	29	5	NO	
ZP	45	M	100	178	31.56	L3-L4	Right	60	15	NO	
ZC	57	F	60	165	22.04	L4-L5	Left	42	4	NO	
LE	57	M	78	173	26.06	L3-L4	Left	75	3	NO	
PA	45	M	70	175	22.86	L3-L4	Right	75	2	NO	
PN	72	M	90	160	35.16	L4-L5	Right	178	13	NO	
BN	48	M	101	185	29.51	L2-L3	Left	99	2	NO	
SD	58	M	75	175	24.49	L4-L5	Right	73	15	NO	
MV	82	M	100	167	35.86	L3-L4	Right	50	0.5	NO	
CM	75	M	65	163	24.46	L5-S1	Left	220	5	YES	5

ID PAT	Junctional herniation	Junctional herniation time (months)	Pre OP deficit	Deficit pre OP STAT NO = 0, YES = 1	DEFICIT RECOVERY	Recovery NO = 0, YES FULL = 1, YES PARTIAL = 2	Pre OP pain radicular pain	Pre OP pain-radicular pain (NRS)	Post OP radicular pain		Post OP PAIN NO = 0, YES decreased = 1, YES unchanged = 2, low back pain = 3
AA	NO		NO	0			YES	10	NO	0	0
BD	NO		YES (left thigh adduction 4+/5)	1	YES full	1	YES	8	NO	0	0
BR	NO		YES (thigh strength deficit)	1	YES full	1	YES	8	NO	0	0
BAM	NO		YES (left anterior tibial deficit + left thigh strength deficit 4+/5)	1	YES full	1	YES	8	NO	0	0
BL	NO		YES (left anterior tibial deficit (4/5))	1	YES full	1	YES	9	NO	0	0
BM	NO		YES (thigh strength deficit)	1	YES full	1	YES	9	NO	0	0
BG	NO		YES (thigh strength deficit)	1	YES full	1	YES	9	NO	0	0
CS	NO		NO	0			YES	10	NO	0	0
CG	NO		YES (right tibial anterior deficit)	1	YES full	1	YES	10	NO	0	0
CC	NO		NO	0			YES	10	NO	0	0
CS	NO		YES (thigh strength deficit)	1	YES full	1	YES	10	NO	0	0
CR	NO		NO	0			YES	8	NO	0	0
FS	NO		YES (thigh strength deficit)	1	YES full	1	YES	6	NO	0	0
FE	NO		YES (thigh strength deficit)	1	YES full	1	YES	6	NO	0	0
GR	NO		YES (thigh strength deficit)	1	YES full	1	YES	8	NO	0	0
GE	YES	33	NO	0			YES	8	NO	0	0
GD	NO		NO	0			YES	8	NO	0	0
GI	NO		NO	0			YES	9	NO	0	0
GI1	NO		YES (thigh strength deficit + left tibial anterior deficit)	1	YES full	1	YES	6	NO	0	0
LF	NO		YES (thigh strength deficit)	1	YES partial	2	YES	9	NO	0	0
LL	NO		YES (right thigh strength deficit)	1	YES full	1	YES	9	NO	0	0
LG	NO		NO	0			YES	9	NO	0	0
MM	NO		YES (thigh strength deficit)	1	YES full	1	YES	8	NO	0	0
MM1	NO		NO	0			YES	10	NO	0	0
MN	NO		YES (left thigh strength deficit)	1	YES partial	2	YES	7	NO	0	0
MMG	NO		NO	0			YES	7	NO	0	0
MD	NO		NO	0			YES	7	NO	0	0
MD	NO		NO	0			YES	8	NO	0	0
MS	NO		YES (right thigh strength deficit)	1	YES partial	2	YES	8	NO	0	0
MA	NO		YES left hip flexion deficit	1	YES full	1	YES	8	NO	0	0
MD	NO		YES (left anterior tibial deficit)	1	YES full	1	YES	8	NO	0	0
MC	NO		YES (left anterior tibial deficit)	1	YES full	1	YES	9	NO	0	0
NE	NO		YES (right thigh strength deficit)	1	YES full	1	YES	9	NO	0	0
NC	NO		YES (right tibialis anterior deficit)	1	YES partial	2	YES	9	NO	0	0
NG	NO		YES (left anterior tibialis deficit)	1	YES partial	2	YES	6	NO	0	0
PS	NO		NO	0			YES	9	NO	0	0
PM	NO		NO	0			YES	9	NO	0	0
PG	NO		NO	0			YES	9	NO	0	0
PA	NO		YES (left thigh strength deficit)	1	YES full	1	YES	9	NO	0	0
RL	NO		NO	0			YES	7	NO	0	0
RS	NO		YES (right thigh strength deficit)	1	YES full	1	YES	9	NO	0	0
RD	NO		YES (deficit in left foot flexion)	1	YES full	1	YES	10	NO	0	0
SE	NO		YES (left tibialis anterior deficit)	1	YES full	1	YES	10	NO	0	0
SE	NO		YES (right thigh strength deficit)	1	YES full	1	YES	8	NO	0	0
SM	NO		YES (right tibialis anterior deficit)	1	YES full	1	YES	8	NO	0	0
SS	NO		YES (left tibialis anterior deficit)	1	YES full	1	YES	8	NO	0	0
SR	NO		YES (right tibialis anterior deficit)	1	YES full	1	YES	9	NO	0	0
SG	NO		YES (right thigh strength deficit)	1	YES full	1	YES	9	NO	0	0
SM	NO		YES (right thigh strength deficit)	1	YES full	1	YES	9	NO	0	0
TL	NO		YES (right thigh strength deficit)	1	YES full	1	YES	7	NO	0	0
TG	NO		NO	0			YES	8	NO	0	0
TL1	NO		NO	0			YES	8	NO	0	0
TE	NO		YES left hip flexion deficit	1	YES full	1	YES	9	NO	0	0
VF	NO		NO	0			YES	9	NO	0	0
ZF	NO		NO	0			YES	9	NO	0	0
ZE	NO		NO	0			YES	7	NO	0	0
ZI	NO		NO	0			YES	9	NO	0	0
GL	NO		NO	0			YES	9	NO	0	0
BM	NO		YES (left thigh strength deficit)	1	YES full	1	YES	8	NO	0	0
YT	NO		YES (right thigh strength deficit)	1	YES full	1	YES	8	NO	0	0
ZM	NO		YES (right tibialis anterior deficit)	1	YES partial	2	YES	8	NO	0	0
FA	NO		NO	0			YES	8	NO	0	0
SM	NO		NO	0			YES	8	NO	0	0
SL	NO		NO	0			YES	10	NO	0	0
ZR	NO		YES (right thigh strength deficit)	1	YES full	1	YES	6	NO	0	0
VR	NO		YES (left thigh strength deficit)	1	YES full	1	YES	9	NO	0	0
GV	NO		YES (left thigh strength deficit)	1	YES full	1	YES	8	NO	0	0
CM	NO		NO	0			YES	8	NO	0	0
CP	YES	30	YES (right thigh strength deficit)	1	YES full	1	YES	9	Low back pain	Low back pain	3
FR	NO		YES (right tibialis anterior deficit)	1	YES full	1	YES	9	Low back pain	Low back pain	3
GS	NO		YES (right tibialis anterior deficit)	1	YES partial	2	YES	9	Low back pain	Low back pain	3
MG	NO		NO	0			YES	9	Low back pain	Low back pain	3
BP	NO		NO	0			YES	7	Low back pain	Low back pain	3
CR	NO		YES right hip flexion deficit	1	YES full	1	YES	8	Low back pain	Low back pain	3
CC	NO		YES (right thigh strength deficit)	1	YES full	1	YES	8	NO	0	0
BR	NO		YES right hip flexion deficit	1	YES full	1	YES	9	NO	0	0
BF	NO		NO	0			YES	9	NO	0	0
PG	NO		YES (right thigh strength deficit)	1	YES full	1	YES	9	NO	0	0
DM	NO		YES (left thigh strength deficit)	1	YES full	1	YES	7	NO	0	0
CGL	NO		NO	0			YES	9	NO	0	0
BI	NO		NO	0			YES	9	YES	6	1
DL	NO		NO	0			YES	8	Low back pain	Low back pain	3
FG	NO		NO	0			YES	8	YES	5	1
FV	NO		YES (left anterior tibialis deficit)	1	YES partial	2	YES	8	YES	4	1
SL	NO		YES (right thigh strength deficit)	1	YES full	1	YES	8	Low back pain	Low back pain	3
BM	NO		YES (right tibialis anterior deficit)	1	NO	0	YES	8	YES	3	1
PG	NO		YES (right thigh strength deficit)	1	NO	0	YES	10	YES	5	1
SL	NO		NO	0			YES	6	YES	3	1
PG	NO		YES right hip flexion deficit	1	YES partial	2	YES	9	Low back pain	Low back pain	3
CA	NO		NO	0			YES	8	Low back pain	Low back pain	3
VM	NO		NO	0			YES	8	NO	0	0
CA	NO		YES (right thigh strength deficit)	1	NO	0	YES	9	YES unchanged	9	2
IC	NO		NO	0			YES	9	YES	3	1
CA	NO		NO	0			YES	9	YES	4	1
CF	NO		NO	0			YES	9	YES	2	1
RE	YES	10	YES (right thigh strength deficit)	1	YES partial	2	YES	10	YES	4	1
VA	NO		NO	0			YES	9	YES	3	1
AMA	NO		YES (right tibialis anterior deficit)	1	YES full	1	YES	9	YES	4	1
FP	NO		NO	0			YES	7	NO	0	0
SR	NO		YES (right tibialis anterior deficit + right thigh strength deficit)	1	YES partial	2	YES	7	Low back pain	Low back pain	3
SO	NO		YES (left tibialis anterior deficit)	1	YES full	1	YES	9	Low back pain	Low back pain	3
DR	NO		YES (right tibialis anterior deficit)	1	YES full	1	YES	8	Low back pain	Low back pain	3
ZE	NO		YES (right tibialis anterior deficit)	1	YES full	1	YES	6	NO	0	0
GB	NO		YES (right thigh strength deficit)	1	YES full	1	YES	8	NO	0	0
RMA	NO		YES (left thigh strength deficit)	1	YES full	1	YES	9	YES	3	1
MR	NO		YES (left tibialis anterior deficit)	1	YES full	1	YES	8	YES	5	1
SB	NO		YES (right thigh strength deficit)	1	YES full	1	YES	8	NO	0	0
PV	NO		YES (right tibialis anterior deficit)	1	YES full	1	YES	8	YES	4	1
CA	NO		YES (deficit in right foot flexion)	1	YES partial	2	YES	8	NO	0	0
GV	NO		YES right hip flexion deficit	1	YES full	1	YES	8	YES	5	1
AN	NO		YES (left anterior tibial deficit + left thigh strength deficit )	1	YES full	1	YES	9	YES unchanged	9	2
FM	NO		YES (right thigh strength deficit)	1	YES full	1	YES	9	YES	3	1
DAA	NO		YES (left tibialis anterior deficit)	1	YES full	1	YES	9	YES	4	1
PA	NO		YES (right tibialis anterior deficit)	1	YES partial	2	YES	9	NO	0	0
ZD	NO		YES (left thigh strength deficit)	1	YES full	1	YES	10	NO	0	0
GI1	NO		YES (left thigh strength deficit)	1	YES full	1	YES	9	NO	0	0
PF	NO		YES (left anterior tibial deficit + left thigh strength deficit )	1	YES full	1	YES	9	YES	4	1
CV	NO		YES (right tibialis anterior deficit)	1	YES full	1	YES	7	NO	0	0
FAM	NO		YES (right tibialis anterior deficit)	1	YES full	1	YES	7	YES	3	1
GM	NO		YES (right thigh strength deficit)	1	YES full	1	YES	9	NO	0	0
AG	NO		YES (right thigh strength deficit)	1	YES full	1	YES	8	NO	0	0
BG	NO		YES (right thigh strength deficit)	1	YES partial	2	YES	6	NO	0	0
VC	NO		YES (right thigh strength deficit)	1	YES full	1	YES	8	YES	4	1
GR	NO		YES (right tibialis anterior deficit + right thigh strength deficit)	1	YES partial	2	YES	9	YES	3	1
ZC	NO		YES (left thigh strength deficit)	1	YES full	1	YES	8	NO	0	0
FF	NO		YES (deficit in left foot flexion)	1	YES full	1	YES	8	NO	0	0
ZP	NO		NO	0			YES	8	NO	0	0
ZC	NO		YES (left thigh strength deficit)	1	YES full	1	YES	8	NO	0	0
LE	NO		YES (left thigh strength deficit)	1	YES partial	2	YES	8	Low back pain	Low back pain	3
PA	NO		YES (right thigh strength deficit)	1	YES partial	2	YES	8	YES	3	1
PN	NO		YES (right thigh strength deficit)	1	YES full	1	YES	9	NO	0	0
BN	NO		YES left hip flexion deficit	1	YES full	1	YES	9	NO	0	0
SD	NO		NO	0			YES	9	NO	0	0
MV	NO		YES (right tibialis anterior deficit + right thigh strength deficit)	1	YES partial	2	YES	7	NO	0	0
CM	NO		YES (right tibialis anterior deficit)	1	YES full	1	YES	10	NO	0	0

Indication for surgery were persistent radicular pain after 4–6 weeks of unsuccessful conservative and medical treatment and/or presence of nerve root palsy. Contraindications to surgery were active cardiovascular disease (acute heart insufficiency, recent myocardial infarction, unstable coronary syndrome) and other contraindications to general anesthesia (i.e., pneumonia, and sepsis).

All patients underwent preoperative MRI and were evaluated for motor recovery and persistence of pain at regular intervals until the third year after discharge. In the event that the patient reported persistence of pain with the same characteristics as preoperatively or the onset of new low back pain with sciatalgic radiations, a new MRI was recommended in order to highlight recurrence and/or the onset of junctional herniation.

### Statistical analysis

The statistical analyses were carried out using the Statistical Package for the Social Sciences, a software package for Windows (version 11.0.1; SPSS, Inc.) (Microsoft Corporation, One Microsoft Way Redmond, WA 98052-7329, USA).

Univariate analysis (Fisher exact test) included impact of age (range 25–86 years), sex, level (range L2/L3 to L5/S1), and BMI (range 18–38.5 kg/m [2] median value = 26.8 kg/m [2] ) on outcome variables, i.e., evidence of junctional herniated disc at follow-up and evidence of recurrence of herniated disc at follow-up. Logistic regression analysis was used for defining the impact of the aforementioned variables on outcome variables. Results presenting *p* ≤ 0.05 were considered statistically significant.

## Results

### Demographic characteristics

We enrolled at the beginning 160 patients with purely foraminal herniated disc operated using trans pars microscopic approach at Ferrara University Hospital between January 2015 and January 2020; 25 were lost during the follow-up in the period between the surgery and the outpatient visit. Therefore, we collected a total of 135 patients, 94 males (69.6%) and 41 females (30.4%), with the age range of 25–86 years old and the median age of 61.3 years old.

In 132 out of 135 (97.8%) the BMI was available with a range of 18.0–38.5 kg/m [2] and the median BMI was 26.8 kg/m [2].

### Clinical characteristics

The locations of the LDHs were as follows: 17 patients presented L2-L3 FLDH (12.87%), 39 L3-L4 FLDH (29.54%), 62 L4-L5 FLDH (46.96%), 16 L5-S1 FLDH (12.12%), and 1 had a FLDH at both L3-L4 and L4-L5 level (0.75%). The right side was the most involved (78 patients – 59.01%), while the left side was affected in 57 cases (43.18%), even though one patient had bilateral involvement (0.75%).

Before surgery, 38 patients had symptoms for 1 month or less (28.03%), while 97 had symptoms for more than 1 month (74.24%); median length of symptomatic period was 4.2 months, while the range was 1 week to 94 months. Before surgery, all of 135 patients had radicular pain (100%) and 91 (67.04%) patients presented with a motor deficit: 33 patients had foot dorsiflexion deficit (36.2%), 3 plantar-flexion (3.3%), 54 presented deficit of the thigh strength (59.3%), one had a deficit of the adduction of the thigh (0.76%), and 7 of hip flexion (7.7%). Seven patients had more than one motor deficit.

### Outcomes

There were no major complications (CSF leak or hemorrhage) during or immediately following surgery.

Blood losses were minimal, no patients needed to be transfused after these surgeries; hospitalization times were minimal too with about 24 h on average of hospital stay with mobilization on the first post op day.

Among 135 patients, 6 experienced recurrence at the same level treated (4.4%): 1 patient presented L3/L4 recurrence after 2 weeks of follow-up treated with left laminectomy; 1 patient had right L3/L4 recurrence after 4 months of follow-up and not retreated because of patient’s choice; 1 patient had left L4/L5 recurrence after 18 months retreated with microscopic trans pars approach; one had a recurrence after 5 months; another one after 7 months both re-treated successfully with trans pars approach; and one patient presented contralateral foraminal LDH after 20 months and was successfully treated surgically.

Among 135 patients, 3 had junctional herniation after surgery (2.2%). One of them had two FLDH at L3-L4 and L4-L5 levels and experienced a junctional asymptomatic herniation at L2-L3 level 33 months after surgery objectively demonstrated with an MRI. One patient treated for L4-L5 FLDH presented a symptomatic L3-L4 junctional herniation 30 months after surgery, which was treated successfully with two peri-radicular steroid injections. One patient treated for L3-L4 FLDH presented a junctional L4-L5 herniation 10 months after surgery, demonstrated through MRI and successfully treated with peri-radicular steroid injections.

Among 91 patient that experienced radicular deficit before surgery, 70 patients referred total recovery in daily activities (75.8%), 18 patients referred partial recovery in daily activities (19.8%), and 3 patients referred no recovery in daily activities (3.3%).

All patients (100%) had radicular pain preoperatively (mean NRS 8, range 6–10, Table 1). After surgery, 96 patients referred no pain (71.1%), 14 patients referred low back pain without sciatica (10.4%), 23 referred diminished but residual radicular pain (17 %), and 2 patients (1.5% of the total) referred that the pain had not changed.

The results of the statistical analysis performed showed that the occurrence of junctional herniated disc or recurrent herniated disc were not influenced by the analyzed variables, both at univariate and at multivariate analyses (Tables 2 and 3).

**Table 2:** Table of Fisher exact test (statistically significant *p* value ≤ 0.05)

	Age	Sex	BMI	Level
Recurrent herniation	0.638	0.408	0.879	0.239
Junctional herniation	0.571	0.334	0.519	0.685
Recurrence or junctional	0.528	0.180	0.743	0.640

**Table 3:** Logistic regression results; outcome variable: recurrence

			95% CI per EXP (B)	
Variables	*p* value	OR	Inferior	Superior
Age	0.651	1349	0.331	5870
Sex	0.253	3502	0.408	30,083
BMI (≤24.9)	0.853			
BMI (=25/≤29.9)	0.924	0.928	0.201	4285
BMI (≥30)	0.580	0.523	0.053	5191
Level (L2/L3/−L3/L4)	0.730			
Level (L4/L5)	0.842	0.245	0.245	5622
Level (L5/S1)	0.439	0.303	0.303	15,665

## Discussion

At the authors’ best knowledge, this is the first study specifically analyzing the rate of recurrences at the same level or at a junctional level in patients with FLHD treated by the microsurgical trans pars approach.

Despite the extensive debate in literature as to what is the best approach in the surgical treatment of these conditions and despite its detractors claiming that the trans isthmus technique is burdened by a higher number of recurrences due to the narrow surgical corridor that would not allow optimal control toward the medial side of the herniation, our results show that out of 135 cases analyzed, 6 recurrences occurred at the same level (4,4%) and only 5 of them were retreated with surgery.

Moreover, the rate of junctional herniation after surgery was also low; only 3 cases out of 135 were detected (2.2%) and none of these cases required surgery.

In order to compare our results, we performed a brief literature review in which it emerges, as said before, that nowadays there are no available published studies specifically analyzing recurrence and/or junctional disc herniation rate after this kind of surgery.

The only pertinent results were obtained combining on Pubmed search the terms “foraminal disc herniation AND recurrence” and “foraminal disc herniation OR recurrence”, including studies which analyzed the rate of hernial recurrence after surgery also with far lateral techniques and excluding items of instrumented surgery; papers in which endoscopic techniques were used and articles in other languages than English (see Table 4).

**Table 4:** Brief review of the literature

Authors	Year	Journal	Title	Primary outcome	N. of patients	N. of recurrences	Conclusion	Reference	Observations
Chang SB et al.	2006	*Spine*	Risk factor for unsatisfactory outcome after lumbar foraminal and far lateral microdecompression	To evaluate the risk factors for unsatisfactory outcome.	184	6	Facet preserving microdecompression is an effective method for foraminal and far lateral root compression. However, in cases of double herniation, total facetectomy is preferable.	Chang SB, Lee SH, Ahn Y, Kim JM. Risk factor for unsatisfactory outcome after lumbar foraminal and far lateral microdecompression. Spine (Phila Pa 1976). 2006 May 1;31(10):1163-7. doi: 10.1097/01.brs.0000216431.69359.91. PMID: 16648754.	
Porchet F	1999	*Journal of Neurosurgery*	Long-term follow up of patients surgically treated by the far-lateral approach for foraminal and extraforaminal lumbar disc herniations	To evaluate the long-term benefit in 202 patients who were surgically treated via a microsurgical far-lateral approach for foraminal or extraforaminal lumbar disc herniations.	202	11	The far-lateral approach is a safe, effective procedure that avoids the risk of secondary spinal instability.	Porchet F, Chollet-Bornand A, de Tribolet N. Long-term follow up of patients surgically treated by the far-lateral approach for foraminal and extraforaminal lumbar disc herniations. J Neurosurg. 1999 Jan;90(1 Suppl):59-66. doi: 10.3171/spi.1999.90.1.0059. PMID: 10413127.	
Kotil K et al.	2007	*Journal of Spinal disorders and technique*	A minimally invasive transmuscular approach to far-lateral L5-S1 level disc herniations: a prospective study	To assess the efficacy of a surgical technique that is a minimally invasive intermuscular approach (MIIMA) for decompression of L5-S1 far-lateral level disc herniation (FLLDH).	28	0	The MIIMA procedure provides a simple alternative for treating lumbar foraminal or lateral exit zone herniated discs in selected cases. This approach is effective, allowing the preservation of the L5-S1 facet joint, saving the facet joint, preventing postoperative instability, and offering a direct view of the L5-S1 neuroforamen.	Kotil K, Akcetin M, Bilge T. A minimally invasive transmuscular approach to far-lateral L5-S1 level disc herniations: a prospective study. J Spinal Disord Tech. 2007 Apr;20(2):132-8. doi: 10.1097/01.bsd.0000211268.43744.2a. PMID: 17414982.	
Sasani M et al.	2007	*Minimally Invasive Neurosurgery*	Percutaneous endoscopic discectomy for far lateral lumbar disc herniations: prospective study and outcome of 66 patients	To study the outcome of PED for treatment of foraminal or extraforaminal disc herniation	66	1	Percutaneous endoscopic discectomy is a minimally invasive method and offers many benefits to the patient, but extensive surgical practice is needed to become a capable surgeon. Consequently this technique can only be a treatment option on appropriate patients. This study reconfirmed that the removal of fragmented disc material is achieved and offers a pain-free status	Sasani M, Ozer AF, Oktenoglu T, Canbulat N, Sarioglu AC. Percutaneous endoscopic discectomy for far lateral lumbar disc herniations: prospective study and outcome of 66 patients. Minim Invasive Neurosurg. 2007 Apr;50(2):91-7. doi: 10.1055/s-2007-984383. PMID: 17674295.	
Teli M et al.	2010	*European Spine Journal*	Higher risk of dural tears and recurrent herniation with lumbar micro-endoscopic discectomy	To investigate the hypothesis of different outcomes and complications obtainable with the three techniques.	240	13	Outcome measures are equivalent 2 years following lumbar discectomy with micro-endoscopy, microscopy or open technique, but severe complications are more likely and costs higher with micro-endoscopy.	Teli M, Lovi A, Brayda-Bruno M, Zagra A, Corriero A, Giudici F, Minoia L. Higher risk of dural tears and recurrent herniation with lumbar micro-endoscopic discectomy. Eur Spine J. 2010 Mar;19(3):443-50. doi: 10.1007/s00586-010-1290-4. Epub 2010 Feb 3. PMID: 20127495; PMCID: PMC2899770.	Three techniques: micro-endoscopic, micro, open discectomy
Lübbers T et al.	2012	*Acta Neurochirurgica*	Percutaneous endoscopic treatment of foraminal and extraforaminal disc herniation at the L5-S1 level	To present the outcome of percutaneous endoscopic lumbar discectomy (PELD) of these lateral and far lateral disc herniations at the L5-S1 level using the newly described foraminal retreat technique in a group of patients with similar preoperative diagnostic studies.	22	2	Percutaneous endoscopic discectomy using the foraminal retreat technique is an effective treatment method for patients with foraminal and extraforaminal disc herniations at the L5-S1 level on appropriately selected patients.	Lübbers T, Abuamona R, Elsharkawy AE. Percutaneous endoscopic treatment of foraminal and extraforaminal disc herniation at the L5-S1 level. Acta Neurochir (Wien). 2012 Oct;154(10):1789-95. doi: 10.1007/s00701-012-1432-z. Epub 2012 Jul 11. PMID: 22782651.	
Choi KC et al.	2013	*Pain Physician*	Percutaneous endoscopic lumbar discectomy for L5-S1 disc herniation: transforaminal versus interlaminar approach	To compare the radiologic features and results of TF-PELD and IL-PELD. We have clarified the patient selection for the PELD route for L5-S1 disc herniation.	30	3.3% TF-PELD; 6.7% IL-PELD	This study demonstrated that TF-PELD is preferred for shoulder type, centrally located, and recurrent disc herniation, while IL-PELD is preferred for axillary type and migrated discs, especially those of a high grade.	Choi KC, Kim JS, Ryu KS, Kang BU, Ahn Y, Lee SH. Percutaneous endoscopic lumbar discectomy for L5-S1 disc herniation: transforaminal versus interlaminar approach. Pain Physician. 2013 Nov-Dec;16(6):547-56. PMID: 24284840.	
Yokosuka J et al.	2016	*Journal of Spine Surgery*	Advantages and disadvantages of posterolateral approach for percutaneous endoscopic lumbar discectomy	To focus the posterolateral approach (PLA) and investigate the appropriate operative indication	29	1	PLA can be safely used to treat foraminal and extraforaminal LDH with foraminal height ≥13 mm and foraminal width ≥7 mm. The procedure is effective for preserving the facet joint; however, we should carefully consider the indications when local scoliosis and/or instability are present.	Yokosuka J, Oshima Y, Kaneko T, Takano Y, Inanami H, Koga H. Advantages and disadvantages of posterolateral approach for percutaneous endoscopic lumbar discectomy. J Spine Surg. 2016 Sep;2(3):158-166. doi: 10.21037/jss.2016.09.03. PMID: 27757427; PMCID: PMC5067274.	
Choi KC et al.	2017	*World Neurosurgery*	Usefulness of Percutaneous Endoscopic Lumbar Foraminoplasty for Lumbar Disc Herniation	To evaluate the efficacy of foraminoplasty for HD and propose applicable situations for foraminoplasty in PELD.	136	4 (1 FG, 3 NFG)	Percutaneous endoscopic lumbar foraminoplasty may be effective for small DH, migration, sequestration, recurrent HD, HD in L5-S1 with a high iliac crest, and central HD with a wide lamina angle.	Choi KC, Shim HK, Park CJ, Lee DC, Park CK. Usefulness of Percutaneous Endoscopic Lumbar Foraminoplasty for Lumbar Disc Herniation. World Neurosurg. 2017 Oct;106:484-492. doi: 10.1016/j.wneu.2017.07.035. Epub 2017 Jul 16. PMID: 28720527.	FG: foraminoplasty group; NFG: non foraminoplasty group
De Bonis P et al.	2017	*Spine*	Transpars Microscopic Approach for the Treatment of Purely Foraminal Herniated Lumbar Disc: A Clinical, Radiological, Two-center Study	To assess the safety and efficacy of treating patients with lumbar foraminal disc herniations via a microscopic transpars approach, with a clinical and radiological follow-up evaluation.	47	0	Transpars microscopic approach is effective and safe for the treatment of FLDH.	De Bonis P, Mongardi L, Pompucci A, Ricciardi L, Cavallo MA, Farneti M, Lapparelli M, Capone G, Altruda C, Schivalocchi R, Campioni P, Ghisellini G, Trapella G. Transpars Microscopic Approach for the Treatment of Purely Foraminal Herniated Lumbar Disc: A Clinical, Radiological, Two-center Study. Spine (Phila Pa 1976). 2017 Mar 15;42(6):E371-E378. doi: 10.1097/BRS.0000000000001839. PMID: 27496668.	
Wong KW et al.	2018	*World Neurosurgery*	Clinical Outcome of Minimally Invasive Decompression Without Discectomy in Contained Foraminal Disc Herniation: A Single-Center Study	To evaluate the benefits of stand-alone decompression without discectomy for patients with contained foraminal disc herniation.	17	0	Stand-alone decompression without discectomy is an effective method for relieving symptoms and preserving the disc in contained foraminal disc herniation. A minimally invasive approach with thorough decompression techniques yields good results.	Wong KW, Ho CH, Yu TC, Wu CD, Tsang YS. Clinical Outcome of Minimally Invasive Decompression Without Discectomy in Contained Foraminal Disc Herniation: A Single-Center Study. World Neurosurg. 2018 Oct;118:e367-e374. doi: 10.1016/j.wneu.2018.06.192. Epub 2018 Jun 30. PMID: 29969734.	NOT PERTINENT
Kim HS et al.	2018	*Journal of Visualized Experiments*	A Mobile Outside-in Technique of Transforaminal Lumbar Endoscopy for Lumbar Disc Herniations	To describe the technical aspects of a novel mobile outside-in method in dealing with different types of disc prolapse.	184	15	This article presents a novel outside-in approach that relies on a precise landing within the foramen in a mobile manner and does not solely depend upon the enlargement of the foramen	Kim HS, Adsul N, Kapoor A, Choi SH, Kim JH, Kim KJ, Bang JS, Yang KH, Han S, Lim JH, Jang JS, Jang IT, Oh SH. A Mobile Outside-in Technique of Transforaminal Lumbar Endoscopy for Lumbar Disc Herniations. J Vis Exp. 2018 Aug 7;(138):57999. doi: 10.3791/57999. PMID: 30148483; PMCID: PMC6126677.	NOT PERTINENT
Bae JS et al.	2018	*Neurocirurgia*	Extreme lateral and interlaminar approach for intra-canal and foraminal double disc herniation at lumbosacral level	To compare the approach with the conventional combined interlaminar and paraisthmic approach (CIPA).	35	4 (CIPA group)	In the treatment of L5-S1 double disc herniation, the ELIA surgical approach showed better outcomes than the CIPA surgical approach did with respect to pain and K-ODI during a mid-term follow-up examination conducted three months post-operation.	Bae JS, Kim KJ, Kang MS, Jang IT. Extreme lateral and interlaminar approach for intra-canal and foraminal double disc herniation at lumbosacral level. Neurocirugia (Astur : Engl Ed). 2019 Mar-Apr;30(2):53-59. English, Spanish. doi: 10.1016/j.neucir.2018.07.002. Epub 2018 Sep 28. PMID: 30274950.	Article in Spanish
Zhang Y	2018	*Quantitative Imaging in medicine and Surgery*	The modified transforaminal endoscopic technique in treating intracanalicular combining foraminal and/or extraforaminal lumbar disc herniations	To develop a modified transforaminal endoscopic spine system (TESSYS®) technique for treating intracanalicular combining foraminal and/or extraforaminal lumbar disc herniation (ICFE-LDH), and evaluate the technical efficacy and safety.	22	1	The modified TESSYS technique is a minimally-invasive, effective and safe surgery for treating ICFE-LDHs in selected patients	Zhang Y, Pan Z, Yu Y, Zhang D, Ha Y, Yi S, Shin DA, Sun J, Koga H, Phan K, Azimi P, Huang W, Cao K; written on behalf of AME Spine Surgery Collaborative Group. The modified transforaminal endoscopic technique in treating intracanalicular combining foraminal and/or extraforaminal lumbar disc herniations. Quant Imaging Med Surg. 2018 Oct;8(9):936-945. doi: 10.21037/qims.2018.10.02. PMID: 30505722; PMCID: PMC6218206.	New minimally invasive technique to treat endoscopically the foraminal disch herniation
Abdelgawaad AS et al.	2018	*The Spine Journal*	Extraforaminal microscopic assisted percutaneous nucleotomy for foraminal and extraforaminal lumbar disc herniations	To evaluate the clinical outcome, complications recurrence, and reoperation rate of extraforaminal microscopic-assisted percutaneous nucleotomy, with literature review focusing on complications and recurrence rate.	76	2	Trans-tubular percutaneous extraforaminal microscopic-assisted nucleotomy is effective for foraminal and extraforaminal disc herniations. It is a muscle-splitting minimally invasive approach with minimal morbidity. Complications, recurrence, and reoperation rate are not different compared with microsurgical open or endoscopic techniques.	Shawky Abdelgawaad A, Babic D, Siam AE, Ezzati A. Extraforaminal microscopic assisted percutaneous nucleotomy for foraminal and extraforaminal lumbar disc herniations. Spine J. 2018 Apr;18(4):620-625. doi: 10.1016/j.spinee.2017.08.258. Epub 2017 Sep 4. PMID: 28882526.	
LewandrowskiKai-Uwe	2018	*International Journal of Spine Surgery*	Readmissions After Outpatient Transforaminal Decompression for Lumbar Foraminal and Lateral Recess Stenosis	To analyze readmission rates after outpatient transforaminal endoscopic decompression surgery for lumbar foraminal and lateral recess stenosis done in an ambulatory surgery center.	1839	9	Transforaminal endoscopic decompression can be successfully carried out in an outpatient surgery center setting. Readmissions due to reherniations, postoperative complications, or poor pain control are uncommon.	Lewandrowski KU. Readmissions After Outpatient Transforaminal Decompression for Lumbar Foraminal and Lateral Recess Stenosis. Int J Spine Surg. 2018 Aug 15;12(3):342-351. doi: 10.14444/5040. PMID: 30276091; PMCID: PMC6159758.	
Park CH et al.	2019	*Pain Physician*	Risk Factors for Early Recurrence After Transforaminal Endoscopic Lumbar Disc Decompression	To identify factors correlating with early HLD recurrence after TELD.	1900	209	In patients undergoing TELD procedures, smaller-sized herniated discs are linked to early recurrences.	Park CH, Park ES, Lee SH, Lee KK, Kwon YK, Kang MS, Lee SY, Shin YH. Risk Factors for Early Recurrence After Transforaminal Endoscopic Lumbar Disc Decompression. Pain Physician. 2019 Mar;22(2):E133-E138. PMID: 30921991.	Recurrences were unrelated to gender, BMI, DM or HTN, smoking status, migration grade, nature (Dht or Dbase of herniated disc), or the presence of spondylolisthesis.
Kim HS et al.	2019	*BioMed Research International*	Predictive Scoring and Risk Factors of Early Recurrence after Percutaneous Endoscopic Lumbar Discectomy	To predict the early recurrence after full endoscopic lumbar discectomy, we analyzed factors related to demographic factor anatomical factors, operative method, and postoperative management, and predicted the possibility of recurrence according to the scoring system.	300	9.33% (11% A, 10% B, 7% C)	Early recurrence after PELD is associated with several risk factors such as BMI, degeneration scale, combined HNP, and early ambulation. If we use the predicting score, we can postulate the occurrence of early recurrence after PELD. Knowing the predictive factors prior to surgical intervention will allow us to decrease the early recurrence rate after PELD.	Kim HS, You JD, Ju CI. Predictive Scoring and Risk Factors of Early Recurrence after Percutaneous Endoscopic Lumbar Discectomy. Biomed Res Int. 2019 Nov 7;2019:6492675. doi: 10.1155/2019/6492675. PMID: 31828113; PMCID: PMC6881637.	Group A: transforaminal inside-out approach; Group B: transforaminal outside-in approach; Group C: interlaminar approach)
De Bonis P et al.	2020	*Journal of Neurosurgical Sciences*	Transpars approach for L5-S1 foraminal and extra-foraminal lumbar disc herniations: technical note	To determine the feasibility, efficacy and safety of the transpars microscopic approach for the treatment of L5-S1 foraminal and extraforaminal lumbar disc herniation.	14	0	The trans pars microscopic approach is feasible, safe and effective for L5-S1 foraminal and extraforaminal disc herniation. During surgery, the key-point is the oblique working angle, directed caudally, parallel to L5 pedicle. The iliac crest does not seem to constitute an obstacle.	De Bonis P, Musio A, Mongardi L, Lofrese G, La Marca F, Visani J, Cavallo MA, Scerrati A. Transpars approach for L5-S1 foraminal and extra-foraminal lumbar disc herniations: technical note. J Neurosurg Sci. 2020 Dec 9. doi: 10.23736/S0390-5616.20.05165-6. Epub ahead of print. PMID: 33297610.	
Tanaka M et al.	2021	*Journal of Spine Surgery*	Clinical Outcomes and Postoperative Radiographic Assessment of Osteoplastic Hemilaminectomy in the Treatment of Lumbar Foraminal Nerve Root Compression	To review the radiographic and clinical outcomes of osteoplastic hemilaminectomy for the treatment of lumbar foraminal nerve root compression.	51	3	Ninety-four and one percent of the patients who underwent osteoplastic hemilaminectomy achieved a significant improvement in the clinical outcomes and did not require additional surgery within 2 years following the procedure. Over a 5-year follow-up on average, 5.9% of the subjects developed postoperative lumbar segmental instability	Tanaka M, Kanayama M, Hashimoto T, Oha F, Shimamura Y, Endo T, Tsujimoto T, Hara H, Hasegawa Y, Nojiri H, Ishijima M. Clinical Outcomes and Postoperative Radiographic Assessment of Osteoplastic Hemilaminectomy in the Treatment of Lumbar Foraminal Nerve Root Compression. Spine Surg Relat Res. 2021 Feb 9;5(6):352-358. doi: 10.22603/ssrr.2020-0203. PMID: 34966860; PMCID: PMC8668207.	
Alhashash M et al.	2022	*Archives of Orthopedic and Trauma Surgery*	Extra-laminar microscopic-assisted percutaneous nucleotomy (EL-MAPN) for the treatment of foraminal lumbar disc prolapse, a modified minimally invasive approach	In this work, a modification of the percutaneous surgical approach for removing the lumbar foraminal disc prolapse is introduced.	50	2	EL-MAPN represents a minimally invasive approach for foraminal disc prolapse removal under direct visual control avoiding injury to the facet joint or pars interarticularis.	Alhashash M, Gendy H, Shousha M. Extra-laminar microscopic-assisted percutaneous nucleotomy (EL-MAPN) for the treatment of foraminal lumbar disc prolapse, a modified minimally invasive approach. Arch Orthop Trauma Surg. 2022 Oct;142(10):2405-2411. doi: 10.1007/s00402-021-03846-8. Epub 2021 Mar 7. PMID: 33677658.	

Porchet [8] et al. published in 1999 results about 202 patients operated with far lateral technique (187 trans muscular, 15 para muscular). Only 9 out of 202 developed recurrence at the same level (4 reoperated with far lateral technique, 5 with standard interlaminar technique given paramedian recurrence) with a calculated recurrence rate of 4.45 %.

Kotil et al in 2007 [13] published a paper analyzing 14 patients with foraminal/extra foraminal L5/S1 disc herniations treated with trans-muscular technique in which they reported no postoperative recurrence.

In 2018, resuming a technique already proposed in 2003 by Greiner-Perth [14], Abdelgawaad and colleagues [15] performed 76 surgeries with microscopic assisted percutaneous nucleotomy technique for foraminal and extraforaminal lumbar disc herniations obtaining recurrences in only two treated cases (2.63 % of recurrence rate).

A more recent modification of the technique proposed by Abdelgaawad was developed in 2021 by Alhashash. According to this modification, 50 patients with herniated foraminal discs were treated obtaining only 2 recurrences (4%) [16].

A Japanese 2021 study proposed an alternative technique for the treatment of lumbar foraminal pathologies involving hemilaminectomy with laminoplasty, however, this technique was only used in 4 patients with extraforaminal disc herniations and in 44 patients with lumbar herniations not specifying how many of these were foraminal and how many were paramedian. The recurrence rate was settled at 6.25 % (3 recurrent disc hernias out of 48 treated) and in all cases that required re operation a TLIF (transforaminal lumbar inter body fusion) was performed [16]*.*

Except for Porchet’s work in which 2 junctional recurrences are reported and Tanaka’s work in which 1 junctional LDH is reported; no junctional recurrences are detected in the other cited works.

Few years ago De Bonis et al. proposed a different, more conservative approach for the minimally invasive treatment of FLDH that would allow access to the foramen of conjugation only with minimal bony removal at the level of the isthmus: the trans pars interarticularis microscopic approach [17].

Although this approach is not entirely new, it has long been criticized both because of its learning curve and because, in the eyes of its detractors, for the possible difficulty to manage any hernias that extend even medially, potentially thereby promoting the rate of recurrence in the absence, however, of specific literature on the subject.

Results of the present study is intended as a natural complement to the previous published in 2017 adding an analysis of 135 consecutive cases operated with this technique in terms of recurrence rate and the onset of junctional herniations.

Our results show the trans pars approach is comparable to other approaches reported in the current literature (reported by Porchet, Abdelgawaad, and Alhashash, sightly minor comparing it with the Tanaka’s work) in terms of recurrence rate (4.4%) and junctional herniation rate (2.2%).

The trans pars technique is less invasive in terms of muscular damage than, for example, the far lateral trans muscular techniques, using a smaller skin incision, that is comparable to that used for the medial herniectomies (3 cm on average).

It was not in the aim of this paper the analysis of post op instability; we have already published a paper that specifically addressed that issue (De Bonis et al. Spine 2017) [17]. In this series, we did not perform a radiological follow-up, but in patients that clinically presented post-op problems (recurrence of HD, junctional HD, persistent pain). All these patients (39 out of 135 cases) performed MRI and dynamic X-rays, showing no signs of instability and that is because the trans pars technique, as seen, only requires the removal of a very small portion of bone at the histmus level, without touching the articular complex in any way.

All of the above considerations also greatly affect the extremely short surgical times (the average duration of the surgery is about 70 min) as well as a shorter postoperative hospitalization and an earlier return to activities of daily living.

Lastly, the results obtained from statistical analyses show no significant correlations between the rate of LDH recurrence (whether junctional or at the same level) neither with the patient's age nor, more importantly, with BMI.

The latter finding about the non-correlation between BMI and recurrence rate is interesting and in contrast not only with the common thinking, that would like to see a higher probability of recurrences with higher values, but also with some studies published recently in literature although different surgical techniques were used.

As example, Siccoli et al. in 2022 [18] found this correlation statistically significant (*p* = 0.017), the same has done by Wang et al. in 2022 [19] that found a significant correlation both with BMI and patient age (BMI *p* = 0.001, age *p* < 0.001) while Li et al. [20] showed that only a BMI above 25 is somehow related to an increased risk of recurrence considering a wide population sample but operated with percutaneous endoscopic technique.

Several authors have published their series of patients with foraminal lumbar herniation operated either with the endoscope or with the microscope.

No series directly compare these two approaches. Nonetheless, outcome variables in terms of pain control seem to be similar [17, 21–23].

The population sample analyzed in our study is undoubtedly smaller compared to that reported in previously cited papers but, to date, it appears to be the largest treated with the trans pars microscopic technique so this data, while not reaching statistical significance, are nonetheless important and worthy of future investigation perhaps by enlarging the sample.

Correlations between age and outcome, sex and outcome, and age/sex/ BMI/treated level were also not significant in these analyses (Table 2).

## Conclusion

Trans pars microscopic approach for the treatment of FLDH is effective and safe. Recurrence rate in our series is consistently low and in line with the current literature, as well as the onset of junctional LDH rate. Age, sex, BMI, and level of the herniated disc do not influence the rate of recurrences both at same level and at junctional level.

### Supplementary information

ESM 1 (MP4 294 MB)

## Data Availability

The data in this study are shown in Table 1; further information about the statistics is accessible upon request to the authors.
