# Correction to: Recurrent or junctional lumbar foraminal herniated disc in patients operated with trans pars microscopic approach

**DOI:** 10.1007/s10143-023-02158-2

**Published:** 2023-09-19

**Authors:** Matteo Monticelli, Clarissa Ann Elisabeth Gelmi, Alba Scerrati, Michele Alessandro Cavallo, Pasquale De Bonis

**Affiliations:** Neurosurgery Unit, Department of Translational Medicine and for Romagna, Ferrara University, Ferrara, Italy

**Correction to: Neurosurgical Review**

**https://doi.org/10.1007/s10143-023-02109-x**

The authors regret that the author names that appears in the original article were incorrect. The first and last names were swapped.

The original article has been corrected.

